# Multi-organ transcriptomic profiles and gene-regulation network underlying vibriosis resistance in tongue sole

**DOI:** 10.1038/s41597-024-03651-z

**Published:** 2024-07-24

**Authors:** Quanchao Chen, Xinran Ma, Jie Wang, Meng Shi, Guobin Hu, Songlin Chen, Qian Zhou

**Affiliations:** 1 College of Marine Life Sciences, Ocean University of China, Qingdao, 266003 China; 2 State Key Laboratory of Mariculture Biobreeding and Sustainable Goods, Yellow Sea Fisheries Research Institute, Chinese Academy of Fishery Sciences, Qingdao, Shandong 266071 China; 3 Laboratory for Marine Fisheries Science and Food Production Processes, Qingdao Marine Science and Technology Center, Qingdao, Shandong 266237 China

**Keywords:** Data publication and archiving, Bacterial infection

## Abstract

Vibrio spp. are major pathogens responsible for mortality and disease in various marine aquaculture organisms. Effective disease control and genetic breeding strategies rely heavily on understanding host vibriosis resistance mechanisms. The Chinese tongue sole (*Cynoglossus semilaevis*) is economically vital but suffers from substantial mortalities due to vibriosis. Through continuous selective breeding, we have successfully obtained vibriosis-resistant families of this species. In this study, we conducted RNA-seq analysis on three organs, including liver, spleen and intestine from selected resistant and susceptible tongue soles. Additionally, we integrated these data with our previously published RNA-seq datasets of skin and gill, enabling the construction of organ-specific transcriptional profiles and a comprehensive gene co-expression network elucidating the differences in vibriosis resistance. Furthermore, we identified 12 modules with organ-specific functional implications. Overall, our findings provide a valuable resource for investigating the molecular basis of vibriosis resistance in fish, offering insights into target genes and pathways essential for molecular selection and genetic manipulation to enhance vibriosis resistance in fish breeding programs.

## Background & Summary

Currently, the world is facing the food security issue, and aquaculture plays an increasingly important role in food and nutrition supply. However, infectious diseases, especially vibriosis, occur frequently and cause huge mortality and economic loss in fish aquaculture. Vibriosis, typically caused by systemic bacterial infections from bacteria of the *Vibrio* genus, stands as a significant threat in marine aquaculture. Vibriosis is one of the most common bacterial diseases affecting various aquaculture marine fish. Vibriosis accounts for approximately 66.7% of reported diseases in groupers (Epinephelus spp.), impacting all growth stages and causing mortality rates of up to 50% among the fish^1^. Similarly, in the salmon farming industry, *Vibrio* infections represent the most serious problem to date, leading to losses exceeding USD 100 million in Norway^2^. Similarly, vibriosis has emerged as a significant pathogenic disease, resulting in high mortality rates (50%-70%) in Chinese tongue sole^3^. As in other animal production system, the success and stainability of fish aquaculture largely depends on the control of diseases^4^. A viable and efficient way to prevent the disease outbreaks lies in developing new germplasm and varieties characterized by high disease resistance and productivity. To achieve this, it is crucial to understand the molecular and genetic mechanisms underlying the disease resistance trait.

As known, the disease resistance is a highly complex system, consisting of physical barriers preventing the adhesion and invasion of pathogens, interaction between the pathogens and host, and humoral and cellular immune responses in cells^5^. Previous studies of fish regarding to diseases susceptibility/resistance have focused on identifying immune parameters, genetic architectures, and differences in gene expressions. For example, studies in Atlantic salmon (*Salmo salar* L.)^6^ and rainbow trout (*Oncorhynchus mykiss*)^7^ revealed that the furunculosis resistant fish had higher immune responses such as activity of serum complement and non-alpha(2) mantiprotease. Quantitative trait locus (QTL) mapping and genome-wide association study (GWAS) have identified the genetic architecture and divergent locus/genes in several fish species, such as Atlantic salmon^8^, rainbow trout^9,10^ and catfish^11^ At the gene expression level, the differential transcriptomic responses to pathogenic infections of the resistant and susceptible fish have been presented in specific tissue, such as in skin of Atlantic salmon upon sea lice infection^12^, in spleen of common carp (*Cyprinus carpio*) for CyHV-3 virus infection^13^ and in intestine of the tongue sole after *Shewanella algae* infection^14^ and *Vibrio harveyi* infection^15^. These studies together revealed that transcriptomic responses occur systematically in a variety of tissues and organs, and a number of genes and pathways may contribute to the fish disease resistance. However, very few studies were simultaneously carried out on the gene regulatory patterns of multiple tissues and organs, which limited an in-depth understanding of the molecular regulatory mechanisms that could explain the difference in the disease resistance.

The Chinese tongue sole is an economic-important marine fish in China. In recent years, it has suffered from over 70–90% mortality rate caused by vibriosis, caused by the infection of *Vibrio* pathogens such as *V. harveyi* and *V. anguillarum*. To obtain vibriosis resistant germplasms, we have conducted continuous selective breeding programs for 4-5 generations^3,16^. This practice has breed high resistant germplasms, providing a unique opportunity for study the molecular basis underlying the improvement of the vibriosis resistance in fish. In our previous work, we have demonstrated that the selected resistant and susceptible tongue soles were genetically diverged, and identified several genes that were significantly associated with the vibriosis resistance^17,18^. In addition, we compared the gene profiles between resistant and susceptible families, and identified 653 and 1421 DEGs in gill and skin, respectively^18^.

To obtain a holistic insight into the transcriptional regulation for the vibriosis resistance, here we completed the RNA sequencing of liver, spleen and intestine organs from resistant and susceptible tongue soles, respectively. Integrating our previously published transcriptome data of gill and skin tissues, we found that differentially expressed genes (DEGs) were distinct between different organs. Furthermore, weighted gene correlation network analysis (WGCNA) identified tissue-specific functional modules, pathways and genes correlated with the resistant potential against vibriosis. Integrating the DEGs and WGCNA results, our data disclosed that over 1000 genes in different organs may play roles in resisting vibriosis in tongue soles. This is in line with the results in mammals that a number of genes with large range of immune responses control host defense against foreign organisms^19^. These RNA-Seq datasets can be used to discover the plasticity in gene expressions between the resistant and susceptible fish, and thus remedy the knowledge gaps for understanding the interplay between the genetic divergence and the phenotype variation of vibriosis resistance. In addition, our data provide a valuable resource that have important implications enabling effective genetic improvement of disease resistance, which will largely be beneficial to the resistant variety breeding and disease control in aquaculture.

## Methods

### Ethics statement

This study was carried out in accordance with the recommendations of the Care and Use of Laboratory Animals of the Chinese Academy of Fishery Sciences (Ethics Committee number: YSFRI2019002). The protocol was approved by the Animal Care and Use Committee of the Chinese Academy of Fishery Sciences.

### Selective breeding and sample preparation

The selective breeding of the vibriosis resistant and susceptible families for *C. semilaevis* were performed as previously described^3^. Firstly, the genetic sex of parental fish was identified by a sex-specific AFLP marker^20^. Full-sib families were then constructed by strip spawning and were reared in separate common tanks under a flow-through system with the same rearing conditions. To trace the lineage, we tagged each family with visible implant elastomers and the pedigree information was precisely recorded^3^.

**Fig. 1:**
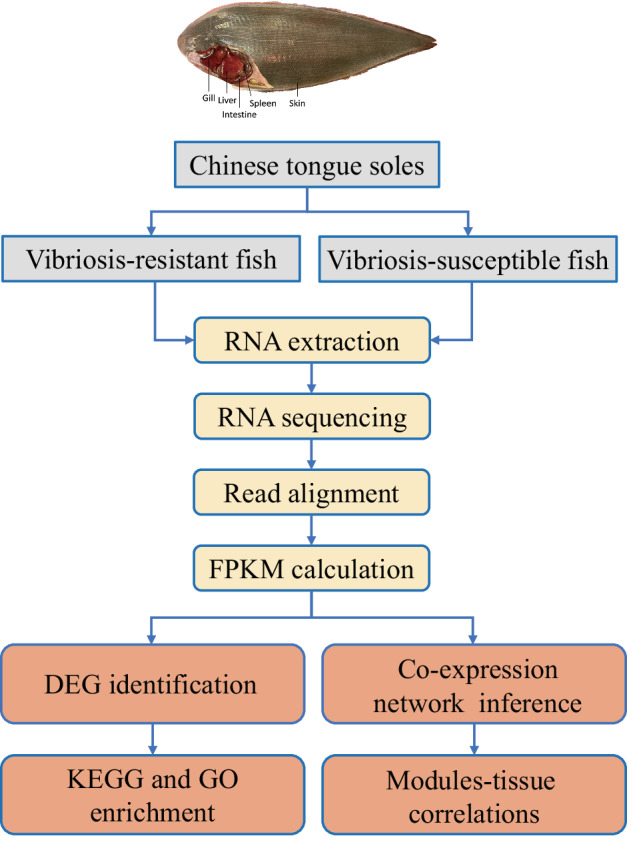
The experimental workflow for resistant tongue sole selection and RNA-Seq data analysis workflows.

In each breeding generation, we performed artificial *Vibrio* challenge tests using the fish with an average size of 10–12 cm. A medial lethal dose (LD_50_) of *V. harveyi* challenge was determined as previously described^3^. After intraperitoneal injection, we recorded the mortality of each family at 12 days post injection, and the families with a survival rate >80% and <30% were considered as *Vibrio* resistant (VR) and susceptible (VS) families, respectively. To date, the artificial challenge and selection for the resistant families processed continuously for more than 12 years, lasting for 5 generations. Fish from the VR and VS families were used for RNA collection (Fig. 1).

### RNA-Seq and comparative transcriptomic analyses

To characterize and compare the gene expression patterns, we collected the liver, spleen and intestine tissues from the resistant and susceptible fish, respectively. Total RNA was extracted from three biological replicates of each group with TRIzol (Invitrogen, USA). Pair-ended (PE) RNA-seq libraries were constructed using the Truseq mRNA stranded RNA-Seq Library Prep Kit (Illumina, USA) according to the standard protocol. Sequencing of totally 18 libraries was conducted with a Illumina HiSeq 2000 sequencing platform, generating raw reads with a read length of 2 × 100 bp and an insert size of 350 bp. The raw data was then quality filtered using RNA-QC-Chain^21^, filtering out the ambiguous N’s, adaptors, low quality reads with more than 20% of the bases having a quality score < 20. Finally, we obtained 62.26–79.69 million raw reads per sample, amounting to a total of 120.35 Gb clean data **(**Table 1). We calculated the fragments per kilobase per million mapped sequence reads (FPKM) value for each gene using RSEM (v1.2.12)^22^, and used NOIseq^23^. to identify the DEGs, with log_2_|FoldChange| ≥ 1 with a probability ≥0.9.

**Table 1:** Summary of the RNA-Seq data for liver, spleen, and intestine from resistant and susceptible families.

Sample	Total raw reads (Mb)	Total clean reads (Mb)	Total clean bases (Gb)	Clean reads ratio (%)	Total mapping ratio
H16_1_In	64.74	62.8	6.25	97	86.59%
H16_2_In	64.75	61.73	6.13	95.33	85.07%
H16_3_In	67.24	62.07	6.17	92.31	89.58%
H16_1_Li	62.26	61.54	6.15	98.85	92.96%
H16_2_Li	62.26	61.29	6.12	98.44	92.12%
H16_3_Li	62.26	61.4	6.13	98.63	89.18%
H16_1_Sp	79.69	78.53	7.83	98.54	89.66%
H16_2_Sp	79.69	78.52	7.8	98.54	87.18%
H16_3_Sp	79.69	78.39	7.81	98.37	88.94%
L47_1_In	64.75	63.02	6.24	97.34	83.95%
L47_2_In	64.75	63.19	6.27	97.6	84.87%
L47_3_In	62.26	60.95	6.05	97.9	84.12%
L47_1_Li	62.26	61.51	6.14	98.8	93.18%
L47_2_Li	62.26	61.26	6.12	98.41	91.99%
L47_3_Li	62.26	61.52	6.14	98.81	91.81%
L47_1_Sp	75.34	74.49	7.39	98.86	87.85%
L47_2_Sp	79.69	78.74	7.8	98.82	88.56%
L47_3_Sp	79.69	78.79	7.81	98.87	88.29%

The clean reads were mapped to the reference genome of the tongue sole (NCBI Accession No. GCA_000523025.1) using BWA^24^ with default parameters. The average mapping rate ranged from 83.95% to 93.18%, averagely at 88.66% (Table 1).

### KEGG and GO enrichment analysis

KEGG and GO enrichment analyses were performed using phyper in R software. KEGG pathways and GO terms with corrected p-values < 0.05 were considered as significantly enriched ones. GO terms are clustered into three main categories: biological processes (BP), molecular functions (MF), and cellular components (CC).

In liver, the central metabolic organ, the 1303 DEGs, including 649 up and 654 down-expressed gene, were enriched in “metabolic pathways” (q < 0.05), covering metabolism processes such as “arginine biosynthesis”, “pyruvate metabolism” and “sphingolipid metabolism” (Table 2).

**Table 2:** The enriched KEGG pathways of the DEGs soles in liver, spleen and intestine between the resistant and susceptible tongue soles.

Organ	KEGG pathway	Qvalue	Pathway ID	Level 1	Level 2
Liver	Metabolic pathways	0.002	ko01100	Metabolism	Global and overview maps
Spleen	Complement and coagulation cascades	3.66E^−18^	ko04610	Organismal Systems	Immune system
	Metabolic pathways	6.53E^−09^	ko01100	Metabolism	Global and overview maps
	Pentose and glucuronate interconversions	1.69E^−06^	ko00040	Metabolism	Carbohydrate metabolism
	Retinol metabolism	1.46E^−05^	ko00830	Metabolism	Metabolism of cofactors and vitamins
	Drug metabolism - cytochrome P450	9.75E^−05^	ko00982	Metabolism	Xenobiotics biodegradation and metabolism
	Metabolism of xenobiotics by cytochrome P450	9.93E^−05^	ko00980	Metabolism	Xenobiotics biodegradation and metabolism
	Ascorbate and aldarate metabolism	1.83E^−04^	ko00053	Metabolism	Carbohydrate metabolism
	Drug metabolism - other enzymes	8.56E^−04^	ko00983	Metabolism	Xenobiotics biodegradation and metabolism
	Ubiquinone and other terpenoid-quinone biosynthesis	1.55E^−03^	ko00130	Metabolism	Metabolism of cofactors and vitamins
	Glycine, serine and threonine metabolism	1.55E^−03^	ko00260	Metabolism	Amino acid metabolism
	Porphyrin and chlorophyll metabolism	2.26E^−03^	ko00860	Metabolism	Metabolism of cofactors and vitamins
	Fat digestion and absorption	2.26E^−03^	ko04975	Organismal Systems	Digestive system
	Peroxisome	6.52E^−03^	ko04146	Cellular Processes	Transport and catabolism
	Steroid biosynthesis	8.43E^−03^	ko00100	Metabolism	Lipid metabolism
	Tyrosine metabolism	9.74E^−03^	ko00350	Metabolism	Amino acid metabolism
	Steroid hormone biosynthesis	0.019	ko00140	Metabolism	Lipid metabolism
Intestine	Pancreatic secretion	0.009	ko04972	Organismal Systems	Digestive system
	Phagosome	0.025	ko04145	Cellular Processes	Transport and catabolism

In spleen, the DEGs were most significantly abundant in “complement and coagulation cascades” (q < 0.05) (Table 2). Interestingly, the 347 up-expressed genes were mainly involved in immune systems, such as “NOD-like receptor signaling pathway” (q < 0.05), “TNF signaling pathway”, “Th1 and Th2 cell differentiation” and “IL-17 signaling pathway”, while the 703 down-expressed genes were significantly enriched in a variety of metabolic pathways. The significantly enriched GO_MF terms for the down-expressed genes were “aromatic amino acid family metabolic process” and “oxidoreductase activity” (q < 0.05).

In intestine, we observed 374 over-expressed and 260 down-expressed genes in the resistant fish. The over-expressed genes were mostly abundant in “pancreatic secretion” pathway (q < 0.05) (Table 2) and GO_MF term of “serine-type endopeptidase activity” (q < 0.05). The down-expressed genes were significantly enriched in “fatty acid metabolism” “phagosome” and “apoptosis”, as well as a GO term of “phospholipid catabolic process” (q < 0.05).

### Weighted gene correlation network analysis (WGCNA)

To gain a holistic and comprehensive gene expression landscape, we used the total 30 RNA-seq datasets, including the newly generated 18 datasets and the previously released 12 datasets, to construct a multiple-organ gene co-expression network.

**Fig. 2:**
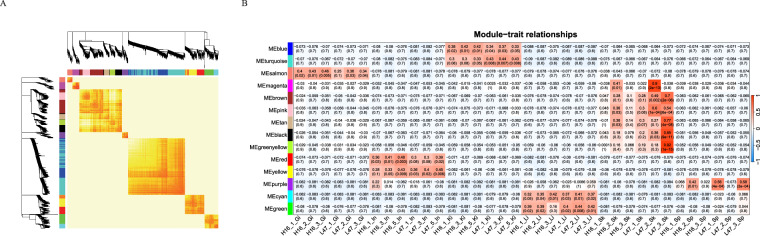
Multiple-tissue gene co-expression network for vibriosis resistance in the tongue sole. (**A**) Hierarchical clustering dendrograms of the co-expressed genes in the modules were identified by WGCNA. A total of 15 modules are colored on the left and top side. The heatmap shows the Person’s association matrix among all genes in the analysis. Yellow color represents low associations and progressively darker red color represents higher associations between genes. (**B**) Associations between co-expressed gene modules and tissue types. Each row corresponds to a module, and each column to a sample. Each cell contains the corresponding correlation with a p-value. The table is color-coded by correlation with intensity and directions of correlations indicated on the right side of the heatmap.

First, we filtered and normalized the genes based on (1) the FPKM value was larger than 1; (2) genes had the FPKM values in more than 16 samples; (3) adjusted the artifacts to make every gene follow an approximate normal distribution. As a result, 20512 genes were used in the normalized expression matrix. Using the mean FPKM values of these genes, we constructed a gene co-expression network and identified the gene co-expression modules with the R package ‘WGCNA’^25,26^. The optimal soft thresholding power of 30 for adjacency computation was graphically determined when the degree of independence was 0.8. Subsequently, the cutreeDynamic function was used for tree pruning of the gene hierarchical clustering dendrograms, resulting in 15 co-expression modules constructed with corresponding Eigengenes (Fig. 2A). Module-sample associations were estimated using the Spearman correlation coefficient analysis between the module eigengenes and tissues type. As a result, we identified 12 modules that were strongly correlated with different tissue types, which was visualized as a heatmap (Fig. 2B). All these tissues might be involved in the determination or regulation of the disease resistance.

**Fig. 3:**
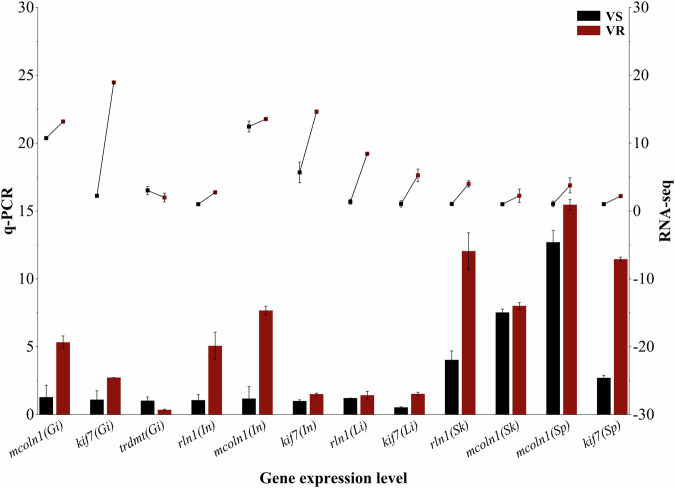
Q-PCR validation of the gene expression levels obtained from RNA-seq. VS: vibriosis susceptible fish, VR: vibriosis resistant fish. Bar height indicates the gene expression level measured by q-PCR (left Y-axis, relative expression measured by ΔΔCt method) and line graph represents RNA-seq measurements (right Y-axis, Fragments Per Kilobase Million, FPKM). X-axis shows gene names with tissues in parentheses.

Genes in the salmon module had a significant correlation with the gill samples and were significantly enriched in KEGG pathways of “nitrogen metabolism” and “proximal tubule bicarbonate reclamation” (*q* < 0.05), as well as GO_MF terms of “ammonium transmembrane transporter activity” (*q* < 0.05).

The purple module had a correlation with spleen. Genes in this module were overrepresented in the digestive system, such as “pancreatic secretion”, “protein digestion and absorption” and “fat digestion and absorption” (*q* < 0.05), as well as GO_MF terms of a series of “peptidase activity” (*p* < 0.05).

The cyan and green modules, which were strongly correlated with the liver tissue, were enriched in the KEGG pathways of “complement and coagulation cascades” and “phagosome” (*q* < 0.05).

The modules in the red and yellow color were correlated with intestine. Genes in these modules were significantly enriched for “glycosphingolipid biosynthesis”, “endocrine, renin-angiotensin system” and “mineral absorption” pathways (*q* < 0.05).

Genes in the brown, pink, tan, black, magenta and the greenyellow modules were correlated with skin. Among them, the brown, pink, tan and black modules were enriched in a number of cellular community pathways, such as “focal adhesion”, “tight junction”, and “adherens junction” (*q* < 0.05). The greenyellow module was mainly enriched for “cardiac muscle contraction” and signal transduction pathways, such as “calcium signaling pathway” and “cGMP-PKG signaling pathway” (*q* < 0.05).

Overall, our results suggest that resistance to *Vibrio* involves a multifaceted response encompassing metabolic adaptations, immune signaling, and tissue-specific gene expression patterns. These findings have implications for understanding the molecular basis of resistance and can inform the development of breeding strategies aimed at enhancing disease resistance in aquaculture species.

## Data Records

The raw data generated from Illumina HiSeq 2000 sequencing platform have been deposited in the NCBI Sequence Read Archive (SRA) database under the NCBI project (https://www.ncbi.nlm.nih.gov/bioproject/) with an accession number of PRJNA785712^27^.

The gene expression data, including gene count, FPKM values and the DEGs set, have been deposited in the GEO database with an accession number of GSE270251^28^.

The gene expression matrix used for gene co-expression network inference, and gene co-expression network analysis results including genes per module are available at Figshare^29^.

## Technical Validation

To validate the gene expression levels obtained by RNA-Seq method, we carried out qPCR analyses to quantify the mRNA levels of 12 randomly selected genes from different tissues. The consistent results of qPCR and RNA-seq indicated that the RNA-Seq results were reliable for further analyses (Fig. 3).

## Data Availability

The authors declare that no custom code was used.

## References

[1] Ina‐Salwany, M. *et al*. Vibriosis in fish: a review on disease development and prevention. **31**, 3-22 (2019). 10.1002/aah.10045 30246889

[2] Sindermann, C. J. H. M. Disease in marine aquaculture. **37**, 505-530 (1984).

[3] Li, Y. *et al*. Genetic analysis of disease resistance to Vibrio harveyi by challenge test in Chinese tongue sole (Cynoglossus semilaevis). *Aquaculture* **503**, 430–435 (2019). 10.1016/j.aquaculture.2019.01.011

[4] Yáñez, J. M., Houston, R. D. & Newman, S. Genetics and genomics of disease resistance in salmonid species. *Frontiers in genetics* **5**, 415, 10.3389/fgene.2014.00415 (2014). 25505486 10.3389/fgene.2014.00415 PMC4245001

[5] Basset, C., Holton, J., O’Mahony, R. & Roitt, I. Innate immunity and pathogen-host interaction. *Vaccine* **21**(Suppl 2), S12–23, 10.1016/s0264-410x(03)00195-6 (2003). 12763678 10.1016/s0264-410x(03)00195-6

[6] Marsden, M. J., Freeman, L. C., Cox, D. & Secombes, C. J. Non-specific immune responses in families of Atlantic salmon, *Salmo salar*, exhibiting differential resistance to furunculosis. *Aquaculture* **146**, 1–16, 10.1016/S0044-8486(96)01358-0 (1996). 10.1016/S0044-8486(96)01358-0

[7] Michel, C. & Hollebecq, M. G. Independence of phagocytic activity and susceptibility to furunculosis in families of rainbow trout (*Oncorhynchus mykiss*) genetically selected for differential resistance. *Fish Shellfish Immunol* **9**, 81–93, 10.1006/fsim.1998.0178 (1999). 10.1006/fsim.1998.0178

[8] Houston, R. D. *et al*. Detection and confirmation of a major QTL affecting resistance to infectious pancreatic necrosis (IPN) in Atlantic salmon (*Salmo salar*). *Developments in biologicals* **132**, 199–204, 10.1159/000317160 (2008). 18817302 10.1159/000317160

[9] Palti, Y. *et al*. Detection and validation of QTL affecting bacterial cold water disease resistance in rainbow trout using restriction-site associated DNA sequencing. *PloS one* **10**, e0138435, 10.1371/journal.pone.0138435 (2015). 26376182 10.1371/journal.pone.0138435 PMC4574402

[10] Vallejo, R. L. *et al*. Similar genetic architecture with shared and unique quantitative trait loci for bacterial cold water disease resistance in two rainbow trout breeding populations. *Frontiers in genetics* **8**, 156, 10.3389/fgene.2017.00156 (2017). 29109734 10.3389/fgene.2017.00156 PMC5660510

[11] Geng, X. *et al*. A genome-wide association study in catfish reveals the presence of functional hubs of related genes within QTLs for columnaris disease resistance. *BMC genomics* **16**, 196, 10.1186/s12864-015-1409-4 (2015). 25888203 10.1186/s12864-015-1409-4 PMC4372039

[12] Robledo, D., Gutiérrez, A. P., Barría, A., Yáñez, J. M. & Houston, R. D. Gene expression response to sea lice in Atlantic salmon skin: RNA sequencing comparison between resistant and susceptible animals. *Frontiers in genetics* **9**, 287, 10.3389/fgene.2018.00287 (2018). 30123239 10.3389/fgene.2018.00287 PMC6086009

[13] Tadmor-Levi, R. *et al*. Different transcriptional response between susceptible and resistant common carp (*Cyprinus carpio*) fish hints on the mechanism of CyHV-3 disease resistance. *BMC genomics* **20**, 1019, 10.1186/s12864-019-6391-9 (2019). 31878870 10.1186/s12864-019-6391-9 PMC6933926

[14] Han, Z. *et al*. Transcriptome profiling of immune-responsive genes in the intestine of *Cynoglossus semilaevis* Günther challenged with *Shewanella algae*. *Fish Shellfish Immunol* **80**, 291–301, 10.1016/j.fsi.2018.06.007 (2018). 29886138 10.1016/j.fsi.2018.06.007

[15] Xu, H. *et al*. Comparative transcriptome profiling of immune response against *Vibrio harveyi* infection in Chinese tongue sole. *Scientific data* **6**, 224, 10.1038/s41597-019-0231-2 (2019). 31641148 10.1038/s41597-019-0231-2 PMC6805913

[16] Chen, S. L. *et al*. Development and characterization for growth rate and disease resistance of families in half-smooth tongue sole(*Cynoglossus semilaevis*). *J. Fisheries China* **34**, 1789–1794, 10.3724/SP.J.1231.2010.07026 (2010). 10.3724/SP.J.1231.2010.07026

[17] Zhou, Q. *et al*. Genome-wide association mapping and gene expression analyses reveal genetic mechanisms of disease resistance variations in *Cynoglossus semilaevis*. *Frontiers in genetics* **10**, 1167, 10.3389/fgene.2019.01167 (2019). 31824570 10.3389/fgene.2019.01167 PMC6880758

[18] Zhou, Q. *et al*. Genomics and transcriptomics reveal new molecular mechanism of vibriosis resistance in fish. *Frontiers in immunology* **13**, 974604, 10.3389/fimmu.2022.974604 (2022). 36304468 10.3389/fimmu.2022.974604 PMC9592550

[19] Breuer, K. *et al*. InnateDB: systems biology of innate immunity and beyond–recent updates and continuing curation. *Nucleic acids research* **41**, D1228–1233, 10.1093/nar/gks1147 (2013). 23180781 10.1093/nar/gks1147 PMC3531080

[20] Chen, S. L. *et al*. Isolation of female-specific AFLP markers and molecular identification of genetic sex in half-smooth tongue sole (*Cynoglossus semilaevis*). *Marine biotechnology (New York, N.Y.)* **9**, 273–280, 10.1007/s10126-006-6081-x (2007). 17308998 10.1007/s10126-006-6081-x

[21] Zhou, Q., Su, X., Jing, G., Chen, S. & Ning, K. RNA-QC-chain: comprehensive and fast quality control for RNA-Seq data. *BMC genomics* **19**, 144, 10.1186/s12864-018-4503-6 (2018). 29444661 10.1186/s12864-018-4503-6 PMC5813327

[22] Li, B. & Dewey, C. N. RSEM: accurate transcript quantification from RNA-Seq data with or without a reference genome. *BMC bioinformatics* **12**, 323, 10.1186/1471-2105-12-323 (2011). 21816040 10.1186/1471-2105-12-323 PMC3163565

[23] Tarazona, S., García-Alcalde, F., Dopazo, J., Ferrer, A. & Conesa, A. Differential expression in RNA-seq: a matter of depth. *Genome research* **21**, 2213–2223, 10.1101/gr.124321.111 (2011). 21903743 10.1101/gr.124321.111 PMC3227109

[24] Jung, Y. & Han, D. BWA-MEME: BWA-MEM emulated with a machine learning approach. *Bioinformatics (Oxford, England)* **38**, 2404–2413, 10.1093/bioinformatics/btac137 (2022). 35253835 10.1093/bioinformatics/btac137

[25] Langfelder, P. & Horvath, S. Eigengene networks for studying the relationships between co-expression modules. *BMC systems biology* **1**, 54, 10.1186/1752-0509-1-54 (2007). 18031580 10.1186/1752-0509-1-54 PMC2267703

[26] Langfelder, P., Zhang, B. & Horvath, S. Defining clusters from a hierarchical cluster tree: the Dynamic Tree Cut package for R. *Bioinformatics (Oxford, England)* **24**, 719–720, 10.1093/bioinformatics/btm563 (2008). 18024473 10.1093/bioinformatics/btm563

[27] *NCBI Sequence Read Archive* https://identifiers.org/ncbi/insdc.sra:SRP351512 (2022).

[28] *GEO* https://identifiers.org/geo/GSE270251 (2024).

[29] Chen, Q-C. *et al*. Multi-organ transcriptomic profiles and gene-regulation network underlying vibriosis resistance in tongue sole. *figshare*10.6084/m9.figshare.c.7045919.v1 (2024).10.6084/m9.figshare.c.7045919.v139048589

